# First Insights into the Viral Communities of the Deep-sea Anoxic Brines of the Red Sea

**DOI:** 10.1016/j.gpb.2015.06.004

**Published:** 2015-10-31

**Authors:** André Antunes, Intikhab Alam, Marta Filipa Simões, Camille Daniels, Ari J.S. Ferreira, Rania Siam, Hamza El-Dorry, Vladimir B. Bajic

**Affiliations:** 1 Computational Bioscience Research Center (CBRC), Computer, Electrical and Mathematical Sciences and Engineering Division (CEMSE), King Abdullah University of Science and Technology (KAUST), Thuwal 23955-6900, Saudi Arabia; 2 Red Sea Research Center (RSRC), Biological and Environmental Sciences and Engineering Division (BESE), King Abdullah University of Science and Technology (KAUST), Thuwal 23955-6900, Saudi Arabia; 3 Biology Department, American University in Cairo, New Cairo 11835, Egypt

**Keywords:** Viral diversity, Metagenomics, Brine–seawater interface, Caudovirales, Red Sea

## Abstract

The deep-sea brines of the **Red Sea** include some of the most extreme and unique environments on Earth. They combine high salinities with increases in temperature, heavy metals, hydrostatic pressure, and anoxic conditions, creating unique settings for thriving populations of novel extremophiles. Despite a recent increase of studies focusing on these unusual biotopes, their viral communities remain unexplored. The current survey explores four metagenomic datasets obtained from different **brine–seawater interface** samples, focusing specifically on the diversity of their viral communities. Data analysis confirmed that the particle-attached viral communities present in the **brine–seawater interfaces** were diverse and generally dominated by **Caudovirales**, yet appearing distinct from sample to sample. With a level of caution, we report the unexpected finding of Phycodnaviridae, which infects algae and plants, and trace amounts of insect-infecting Iridoviridae. Results from Kebrit Deep revealed stratification in the viral communities present in the interface: the upper-interface was enriched with viruses associated with typical marine bacteria, while the lower-interface was enriched with haloviruses and halophages. These results provide first insights into the unexplored viral communities present in deep-sea brines of the **Red Sea**, representing one of the first steps for ongoing and future sampling efforts and studies.

## Introduction

The development and widespread use of molecular-based methods in environmental microbiology revealed that microbes dominate our planet. Ocean-dwelling bacteria are estimated to outnumber stars in the universe by several orders of magnitude (total numbers are 10^29^ and 10^21^, respectively) [1], with even higher values for viruses. Viruses are the most abundant biological entities on Earth (10^30^ for total number of prokaryotic viruses or phages) [2], and harbor the second greatest biomass, after prokaryotes [3], [4]. Furthermore, they are crucial ecological factors, which affect microbial diversity, population dynamics, and the genomes of their hosts [5]. Their impact extends from influencing microbial evolution, to playing an indirect but significant role in the Earth’s biogeochemical cycles [5], [6], [7].

Despite a historically stronger focus on medically-relevant viruses, recent years novel technologies brought forth increasing activities in the field of environmental virology, with multiple studies centered in marine and aquatic environments, as well as several extreme environments. Such ongoing efforts have led to the discovery and description of multiple new viruses and increased our understanding of viral communities (*e.g.*, [2], [5], [8], [9], [10]). Since viruses lack a shared universal phylogenetic marker such as universal ribosomal DNA (rDNA), genetic diversity of environmental viral communities is increasingly assessed through metagenomic sequencing, which provides more and more information about viral diversity and evolution [11], [12]. Nonetheless, metagenomic data have shown that we have yet to discover the majority of viruses present in the environment: over 70% of the genes in the oceanic viral fraction cannot be associated with known viruses [7]. Furthermore, studies on extreme environments, which include a few metagenomic-based surveys (*e.g.*, [13], [14]), have uncovered that hypersaline environments host the highest viral densities reported for aquatic systems [15], yet still very little is known about them.

The deep-sea brines of the Red Sea include some of the most extreme and inaccessible environments on Earth, combining high salinities with increase in temperature, heavy metals, hydrostatic pressure, and anoxic conditions [16]. The microbiology of these brines received considerable attention in the last few years, with studies using an array of culture-dependent [17], [18], [19], [20], [21] and molecular-based approaches, including metagenomic studies [22], [23], [24], [25], [26], [27], [28], [29], [30], [31], [32], [33]. Nonetheless, the viral communities of these extreme biotopes remain unexplored.

This study makes use of four metagenomic samples, obtained from different brine–seawater interfaces from the Red Sea, providing the first, though partial, insights into the viral diversity and community structure present in these environments.

## Results and discussion

In this study, we used the DMAP’s comparison module, associated taxonomic browsing, and filtering capabilities to explore the viral subset of annotations of four metagenomic samples (AT, DD, KU, and KL) obtained from the brine–seawater interfaces of different deeps in the Red Sea. The resulting taxonomic comparison of these samples showed the relative proportions of bacteria, archaea, and viruses (Table 1). While the number of genes associated with viruses might seem relatively low, it should be noted that our source data refers only to reads recovered from the 0.1-μm fraction (*i.e.*, particle-attached or from infected cells). The ensuing analysis is therefore restricted to only part of the total viral diversity present in these environments.

### General viral diversity

**Figure 1:**
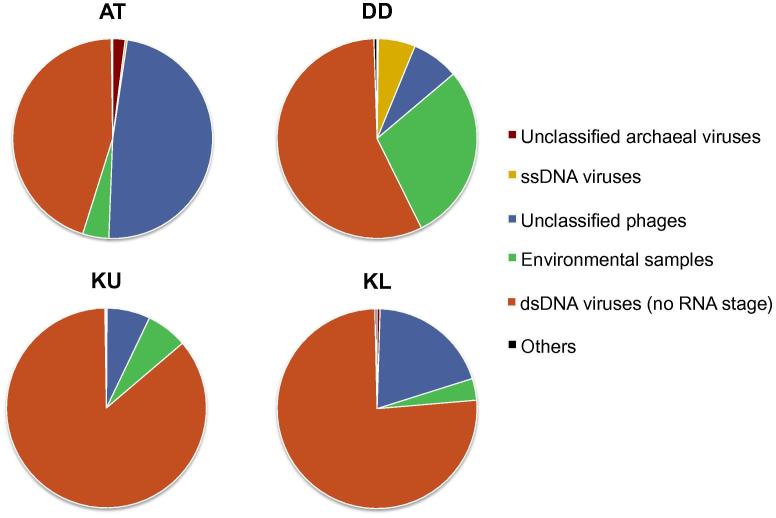
**Relative abundances of members of different viral taxa** Samples were collected on 0.1-μm filters from the brine–seawater interfaces of the Red Sea at different locations. All taxonomical categories mentioned in this study are based on the NCBI Taxonomy database. “Others” include Adenoviridae, Ascoviridae, Baculoviridae, Bicaudaviridae, Fuselloviridae, Herpesvirales, Iridoviridae, Marseilleviridae, Poxviridae, Polydnaviridae, and Salterproviridae. AT, Atlantis II Deep; DD, Discovery Deep; KU, Kebrit Deep upper brine–seawater interface; KL, Kebrit Deep lower brine–seawater interface; dsDNA, double-stranded DNA; ssDNA, single-stranded DNA.

Analysis of the metagenomic datasets confirmed that the particle-attached viral communities present in the brine–seawater interfaces were diverse and, despite some similarities, distinct from sample to sample (Figure 1). This is likely a reflection of differences in microbial community profiles, which are specific to each location, and imparted from changes in physicochemical conditions [16], [26], [30], [31], [32], [33].

**Figure 2:**
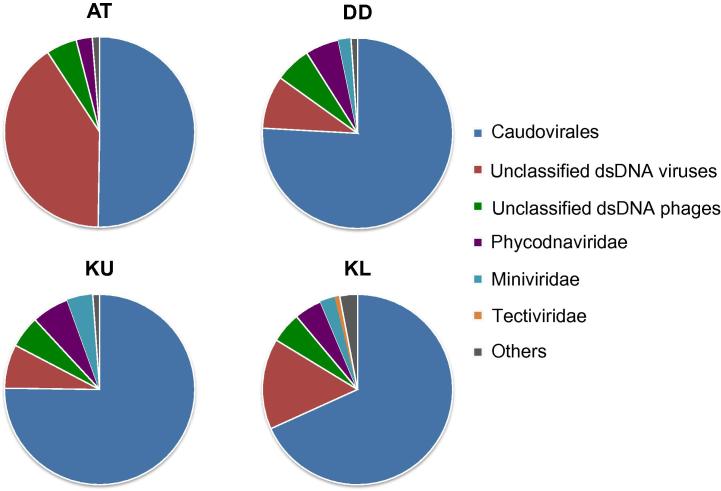
**Relative abundances of double-stranded DNA (dsDNA) viruses** Samples were collected on 0.1-μm filters from the brine–seawater interfaces of the Red Sea at different locations. All taxonomical categories mentioned in this study are based on the NCBI Taxonomy database. “Others” include retro-transcribing viruses, satellites, ssRNA viruses, unassigned viruses, unclassified virophages, and unclassified viruses. AT, Atlantis II Deep; DD, Discovery Deep; KU, Kebrit Deep upper brine–seawater interface; KL, Kebrit Deep lower brine–seawater interface; dsDNA, double-stranded DNA; ssDNA, single-stranded DNA.

As a general trend, we observed a clear dominance of dsDNA viruses, which accounted for 45%–85% of the viruses detected (Figure 1). Further scrutinization indicated that dsDNA viruses are mostly Caudovirales (Figure 2). Caudovirales can be further classified as Syphoviridae, Myoviridae, and Podoviridae, while various proportions of them remained unclassified (Figure S1). Caudovirales are tailed bacteriophages, which are known to dominate in marine and other aquatic environments [9], [34], [35].

Other viral families were detected at much lower abundances, including the unexpected Phycodnaviridae (4%–6%), which infect algae and plants, and trace amounts of insect-infecting Iridoviridae (<1%). These viral taxa are likely derived from the particles originated away from the brines, including host lysis products that act as virus scavengers [5]. We hypothesize that these particles sink through the water column, and are eventually trapped and accumulated in the density gradient of the brine–seawater interface. Viruses (and DNA, in general) have been previously reported as having longer-term stability, and can be preserved in such deep-sea brines [36], [37], [38]. Similar observations have been reported for other marine locations, particularly when transitioning from oxic to anoxic conditions [9]. Detection of these unexpected viral taxa would thus be the result of a “pickling” effect, rather than reflecting the presence of specific hosts in close proximity to the brines.

Samples from Atlantis II Deep had a more divergent profile, with a large, but not dominant proportion of dsDNA viruses (45%) associated with a slightly higher number of unclassified phages (47%; Figure 1). The vast majority of these phages were environmental halophages (data viewable with the taxonomy browser option of DMAP; www.cbrc.kaust.edu.sa/DMAP), which were related to the ones described in a previous study [39]. Furthermore, a significant percentage of dsDNA viruses were also unclassified (40%; Figure 2) and belonged mostly to haloviruses (data viewable with the taxonomy browser option of DMAP; www.cbrc.kaust.edu.sa/DMAP). The unusual combination of very high temperatures and salinities at Atlantis II Deep, which creates one of the harshest environments on Earth and provides a unique prokaryotic host community, might be the main reason behind such high numbers of unclassified viruses. Therefore, one expects that the brine–seawater interface of Atlantis II Deep is a particularly interesting environment for future exploration, with a high potential for discovery of novel viruses infecting polyextremophiles.

### Stratification in viral communities

An additional highlight of our analysis is the stratification of the viral communities within the brine–seawater interface observed for Kebrit Deep. Differences between upper, and lower interface samples from Kebrit Deep were in accordance with the relative position of these layers. Indeed, the upper interface, which is in closer proximity with seawater, was enriched with viruses associated with more typical marine bacteria (*e.g*., *Pelagibacter* and *Synechococcus*), whereas the lower interface, which is closer to the brine, was enriched in haloviruses and halophages (Table 2). Furthermore, such stratification of viral communities is in agreement with previous reports obtained for microbial communities inhabiting brine–seawater interfaces both in the Red Sea and the Mediterranean (*e.g*., [26], [30], [40]).

Although there is some agreement between our results and such previous microbial studies, special care must be taken to avoid inferring direct correlations between microbial and viral communities, due to several well-known limitations and biases. The main caveat of viral metagenomics is that most sequences are unique and thus have no matches in databases [13]. Indeed, while much of the global microbial metagenome has now been sampled, the same cannot be said for the global viral metagenome [11].

Most of our knowledge on viruses still relies heavily on *in vitro* cultured phage–host systems. Surveys of viral diversity are therefore bottlenecked by the lack of environmental isolates with ecological relevance, which frequently evade standard cultivation techniques, resulting in the dominance of culture-independent “unknowns” [41]. Most viral research focuses on strains amenable to laboratorial manipulation, rather than the most relevant or abundant ones [8]. Accordingly, most phage genomes in GenBank are isolated using bacteria from only 3 of the 45 known bacteria phyla (Actinobacteria, Firmicutes, and Gammaproteobacteria), so many others that infect environmental microbes are largely unstudied and unknown [41]. Furthermore, and despite generally being perceived as host-specific predators, information on true host ranges for many viruses is lacking and might be wider than anticipated [5].

### Overview and future work

Despite the aforementioned limitations, results from this study provide important first insights into the unexplored viral communities present in deep-sea brines of the Red Sea and thus represent the first step for ongoing, and future sampling efforts and studies. Future work should circumvent the constraints of this study by including targeted sampling of the viral community (*i.e.*, <0.1 μm fraction) for metagenomic assessment, as well as isolation/characterization, along with studies to determine viral–host dynamics.

## Materials and methods

### Metagenomic samples and DNA sequencing

Metagenomic reads from the four brine–seawater interface libraries were obtained from a previous study (see [22] for further details). Briefly, samples were collected on 0.1-μm filters from the brine–seawater interfaces of Atlantis II Deep, Discovery Deep, and Kebrit Deep, Red Sea. Atlantis II Deep and Discovery Deep are both examples of “hot brines” (with temperatures of 68 °C and 45 °C, respectively), while Kebrit Deep is a colder brine (temperature of 23 °C), which is notorious for its very high sulfur concentration (see [16] for detailed information).

DNA extraction and sequencing were carried out at the American University of Cairo using GS FLX Roche Titanium library guide (see [22] for details).

### Bioinformatics processing of metagenomic reads

Metagenomic data were processed with a focus on viral communities using the Dragon Metagenomic Analysis Platform (DMAP; www.cbrc.kaust.edu.sa/DMAP). Reads were assembled using Newbler software with iterative reference (NCBI RefSeq genomes) and *de novo* assembly procedure, and annotation was carried out using the DMAP annotation module (www.cbrc.kaust.edu.sa/DMAP). Briefly, the module predicts and annotates RNA and protein-coding genes. During annotation, BLAST best hit genes are considered for assigning taxonomic or function information to predicted genes. For taxonomic assignment to RNA genes, NCBI’s small sub-unit (SSU) RNAs and other non-coding RNAs from the European Bioinformatics Institute (EBI) Rfam database are used. For protein-coding genes, the UniProt Knowledgebase (www.uniprot.org), KEGG (www.kegg.jp), eggNOG (http://eggnogdb.embl.de), Conserved Domain Database (CDD, http://www.ncbi.nlm.nih.gov/Structure/cdd/cdd.shtml), and InterPro (http://www.ebi.ac.uk/interpro) databases are used (see associated help provided at DMAP website; www.cbrc.kaust.edu.sa/DMAP). From all the databases described above only archaeal, bacterial, and viral sequences were considered. The following parameters were considered when analyzing these datasets. An *E* value of 1E−3 and BLAST coverage of 50% was considered for BLAST-based analysis, while for other methods, such as InterProscan for domain detection or Infernal for Rfam’s ncRNA predictions, parameters optimized in the source profiles were considered with trusted cutoffs. Annotation results with taxonomic and functional assignments were deposited to the DMAP data warehouse and DMAP comparison module for systematic studies.

Total hit numbers for viral assignments at different taxonomic levels and general statistics for the contigs/singletons are provided as Tables S1 and S2, respectively. All data and analysis tools are openly accessible through the DMAP website at www.cbrc.kaust.edu.sa/DMAP.

## Authors’ contributions

AA conceived, designed, and performed the experiments; AA and MFS analyzed the data; IA, AJSF, RS and HED contributed reagents/materials/analysis tools; AA, IA, MFS, CD, RS, and VBB wrote the manuscript. All authors read and approved the final manuscript.

## Competing interests

The authors have declared no competing interests.

## Figures and Tables

**Table 1:** Breakdown of genes based on taxonomic assignment

**Category**	**Atlantis II Deep**	**Discovery Deep**	**Kebrit Deep (lower)**	**Kebrit Deep (upper)**
Archaea	7102	13,715	58,033	41,752
Bacteria	32,286	84,872	137,482	236,709
Viruses	1210	6498	4781	6499
Total genes	40,598	105,085	200,296	284,960
Percentage of viruses (%)	2.98	6.18	2.39	2.28

**Table 2:** Classification of top ten viral hits based on taxonomic assignment

**Atlantis II Deep**		**Discovery Deep**		**Kebrit Deep lower**		**Kebrit Deep upper**	
**Name**	**%/H**	**Name**	**%/H**	**Name**	**%/H**	**Name**	**%/H**
Halovirus HCTV-5	11.49/139	Uncultured marine virus	18.36/1,193	eHP-28	1.9/91	*Pelagibacter* phage HTVC008M	3.82/248
eHP-12	9.17/111	*Puniceispirillum* phage HMO-2011	5.42/352	Halovirus HGTV-1	1.9/91	Puniceispirillum phage HMO-2011	1.69/110
eHP-23	4.63/56	*Pelagibacter* phage HTVC008M	4.03/262	Halovirus HSTV-1	1.88/90	Moumouvirus	1.48/96
eHP-32	4.63/56	Uncultured virus	2.65/172	Uncultured virus	1.67/80	*Phaeocystis globosa* virus 14T	1.34/87
eHP-31	3.39/41	*Pelagibacter* phage HTVC010P	2.25/146	eHP-23	1.63/78	Uncultured virus	1.34/87
*Bacillus* phage phiNIT1	2.81/34	Marine gokushovirus	2.15/140	eHP-12	1.42/68	*Synechococcus* phage S-SKS1	1.31/85
eHP-6	2.81/34	Halovirus HCTV-5	1.63/106	Halovirus HCTV-5	1.17/56	*Synechococcus* phage S-SM2	1.26/82
eHP-28	2.64/32	Uncultured phage MedDCM-OCT-S05-C113	1.29/84	eHP-10	1.09/52	*Pelagibacter* phage HTVC010P	1.18/77
eHP 1 AAJ-2005	2.56/31	eHP-12	1.25/ 81	eHP-35	1.07/51	*Cafeteria roenbergensis* virus BV-PW1	1.15/75
eHP-11	2.48/30	*Synechococcus* phage S-SM2	1.2/78	*Synechococcus* phage S-SM2	1.02/49	*Sinorhizobium* phage phiM12	1.15/75

*Note:* %, Percentage of total viral hits; H represents total number of hits; eHP, environmental halophage.

## Supplementary material

Supplementary Figure S11 Relative abundances of members of the Caudovirales. Samples were collected on 0.1-μm filters from the brine–seawater interfaces of the Red Sea at different locations. AT, Atlantis II Deep; DD, Discovery Deep; KU, Kebrit Deep upper brine–seawater interface; KL, Kebrit Deep lower brine–seawater interface.

Supplementary Table S1 Breakdown of genes based on taxonomic assignment.

Supplementary Table S2 Classification of top ten viral hits based on taxonomic assignment
