# Supplementary material for: First Insights into the Viral Communities of the Deep-sea Anoxic Brines of the Red Sea
Source: Genomics Proteomics Bioinformatics. 2015 Oct 31;13(5):304–9. doi: 10.1016/j.gpb.2015.06.004 (PMC4678784; doi:10.1016/j.gpb.2015.06.004)
Supplement: Supplementary Figure S11 — Relative abundances of members of the Caudovirales. Samples were collected on 0.1-μm filters from the brine–seawater interfaces of the Red Sea at different locations. AT, Atlantis II Deep; DD, Discovery Deep; KU, Kebrit Deep upper brine–seawater interface; KL, Kebrit Deep lower brine–seawater interface. [file mmc1.pptx]

## Slide 1
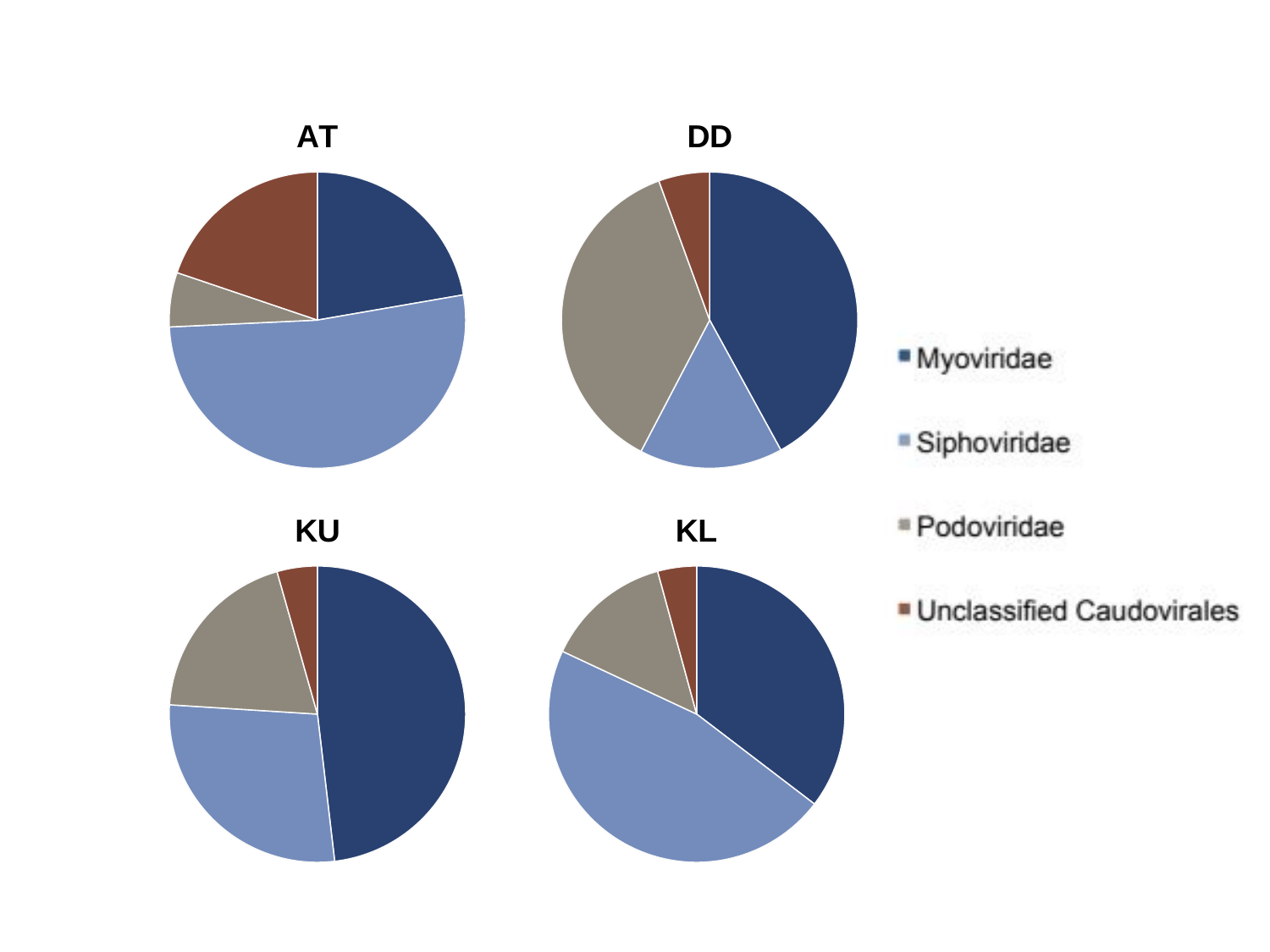

### Chart:
| Category | AT |
|---|---|
| Myoviridae | 45.0 |
| Siphoviridae | 105.0 |
| Podoviridae | 12.0 |
| Unclassified Caudovirales | 40.0 |
### Chart:
| Category | DD |
|---|---|
| Myoviridae | 959.0 |
| Siphoviridae | 357.0 |
| Podoviridae | 839.0 |
| Unclassified Caudovirales | 127.0 |
### Chart:
| Category | KU |
|---|---|
| Myoviridae | 1631.0 |
| Siphoviridae | 944.0 |
| Podoviridae | 664.0 |
| Unclassified Caudovirales | 149.0 |
### Chart:
| Category | KL |
|---|---|
| Myoviridae | 675.0 |
| Siphoviridae | 889.0 |
| Podoviridae | 263.0 |
| Unclassified Caudovirales | 81.0 |
