# Supplementary material for: First Insights into the Viral Communities of the Deep-sea Anoxic Brines of the Red Sea
Source: Genomics Proteomics Bioinformatics. 2015 Oct 31;13(5):304–9. doi: 10.1016/j.gpb.2015.06.004 (PMC4678784; doi:10.1016/j.gpb.2015.06.004)
Supplement: Supplementary Table S1 — Breakdown of genes based on taxonomic assignment. [file mmc2.docx]

**Table S1 Abundance of members of different viral taxa**

| **Viral taxa** | | **Total hits** | | | | | | | |
| --- | --- | --- | --- | --- | --- | --- | --- | --- | --- |
|  |  | **AT** | | **DD** | | **KU** | | **KL** | |
|  |  | **Low** | **High** | **Low** | **High** | **Low** | **High** | **Low** | **High** |
| **Viruses** | Unclassified phages | 574 | 433 | 501 | 404 | 452 | 366 | 927 | 721 |
|  | Unclassified viruses | 1 | 1 | 24 | 20 | 8 | 6 | 12 | 7 |
|  | ssDNA viruses | 5 | 3 | 372 | 317 | 4 | 3 |  |  |
|  | dsDNA viruses (no RNA stage) | 547 | 403 | 3270 | 3006 | 5556 | 4493 | 3632 | 2790 |
|  | Retro-transcribing viruses |  |  | 2 | 2 | 4 | 2 | 2 | 2 |
|  | dsRNA viruses | 1 |  |  |  |  |  |  |  |
|  | Environmental samples | 55 | 38 | 1885 | 1527 | 459 | 349 | 181 | 129 |
|  | ssRNA viruses | 2 | 1 | 4 | 1 | 12 | 4 | 5 | 4 |
|  | Unclassified archaeal viruses | 25 | 18 | 13 | 11 | 2 | 2 | 20 | 18 |
|  | Satellites |  |  | 4 | 1 |  |  |  |  |
|  | Unclassified virophages |  |  | 3 | 3 | 1 |  | 2 |  |
|  | Unclassified viruses |  |  |  |  | 1 | 1 |  |  |
|  | **Subtotals** | **1210** | **897** | **6078** | **5292** | **6499** | **5226** | **4781** | **3671** |
| **dsDNA viruses** | Poxviridae | 1 |  | 21 | 6 | 12 | 11 | 10 | 8 |
|  | Baculoviridae |  |  | 3 | 1 | 8 | 5 | 4 | 4 |
|  | Fuselloviridae | 2 |  | 2 | 2 | 1 | 1 | 23 | 19 |
|  | Polydnaviridae |  |  | 5 | 4 |  |  | 2 | 2 |
|  | Iridoviridae | 4 | 1 | 10 | 7 | 21 | 15 | 12 | 7 |
|  | Phycodnaviridae | 21 | 11 | 208 | 173 | 349 | 287 | 194 | 132 |
|  | Adenoviridae |  |  |  |  | 1 | 1 | 1 |  |
|  | Tectiviridae |  |  | 1 | 1 | 4 | 2 | 30 | 24 |
|  | Corticoviridae |  |  | 1 |  |  |  |  |  |
|  | Caudovirales | 265 | 202 | 2792 | 2282 | 4191 | 3388 | 2477 | 1908 |
|  | Ascoviridae |  |  |  |  | 3 | 2 | 2 | 1 |
|  | Unclassified dsDNA viruses | 217 | 163 | 346 | 272 | 417 | 336 | 552 | 432 |
|  | Unclassified dsDNA phages | 29 | 21 | 231 | 184 | 318 | 244 | 191 | 146 |
|  | Asfarviridae |  |  | 2 |  | 2 |  | 1 |  |
|  | Salterprovirus | 1 | 1 | 4 | 3 | 4 | 3 | 34 | 27 |
|  | Bicaudaviridae | 3 | 3 | 6 | 6 | 2 | 2 | 3 | 3 |
|  | Herpesvirales |  |  | 4 | 1 | 1 | 1 | 6 | 3 |
|  | Mimiviridae | 1 |  | 82 | 63 | 222 | 195 | 84 | 70 |
|  | Marseilleviridae |  |  | 7 | 3 | 20 | 11 | 15 | 10 |
|  | **Subtotals** | **544** | **402** | **3725** | **3008** | **5576** | **4504** | **3641** | **2796** |
| **Caudovirales** | Myoviridae | 70 | 45 | 1156 | 959 | 2006 | 1631 | 900 | 675 |
|  | Siphoviridae | 124 | 105 | 468 | 357 | 1202 | 944 | 1121 | 889 |
|  | Podoviridae | 21 | 12 | 991 | 839 | 802 | 664 | 353 | 263 |
|  | Unclassified Caudovirales | 50 | 40 | 177 | 127 | 181 | 149 | 103 | 81 |
|  | **Subtotals** | **265** | **202** | **2792** | **2282** | **4191** | **3388** | **2477** | **1908** |

*Note*: total hits were obtained using two different cutoffs: low (looser threshold *E* value of 1e−2 and BLAST coverage of 30 %) and high (stricter threshold *E* value of 1e−3 and BLAST coverage of 50 %). AT, Atlantis II Deep; DD, Discovery Deep; KU, Kebrit Deep upper brine-seawater interface; KL, Kebrit Deep lower brine-seawater interface.
