# Supplementary material for: First Insights into the Viral Communities of the Deep-sea Anoxic Brines of the Red Sea
Source: Genomics Proteomics Bioinformatics. 2015 Oct 31;13(5):304–9. doi: 10.1016/j.gpb.2015.06.004 (PMC4678784; doi:10.1016/j.gpb.2015.06.004)
Supplement: Supplementary Table S2 — Classification of top ten viral hits based on taxonomic assignment [file mmc3.docx]

**Table S2 General statistics for viral contigs/singletons**

| **Contigs/singletons** | **AT** | **DD** | **KU** | **KL** |
| --- | --- | --- | --- | --- |
| Total number | 1195 | 6416 | 6447 | 4769 |
| Mean size (bp) | 537.38 | 468.69 | 406.33 | 401.99 |
| Median size (bp) | 439 | 428 | 369 | 390 |
| Longest (bp) | 2144 | 2978 | 3333 | 2099 |
| Shortest (bp) | 200 | 200 | 200 | 200 |
| > 500 nt (hits/ % total) | 493 (41.26 %) | 1366 (21.29 %) | 787 (12.21 %) | 547 (11.47 %) |
| > 1000 nt (hits/ % total ) | 101 (8.45 %) | 234 (3.65 %) | 167 (2.59 %) | 54 (1.13 %) |
| N50 | 613 | 462 | 409 | 417 |

*Note*: AT, Atlantis II Deep; DD, Discovery Deep; KU, Kebrit Deep upper brine-seawater interface; KL, Kebrit Deep lower brine-seawater interface. N50 indicates the length of the smallest contig in the set that contains the fewest (largest) contigs whose combined length represents at least 50% of the assembly.
